# Comparative Analysis of Chloroplast Genomes of Seven *Chaetoceros* Species Revealed Variation Hotspots and Speciation Time

**DOI:** 10.3389/fmicb.2021.742554

**Published:** 2021-11-03

**Authors:** Qing Xu, Zongmei Cui, Nansheng Chen

**Affiliations:** ^1^College of Life Science and Technology, Huazhong Agricultural University, Wuhan, China; ^2^CAS Key Laboratory of Marine Ecology and Environmental Sciences, Institute of Oceanology, Chinese Academy of Sciences, Qingdao, China; ^3^Laboratory of Marine Ecology and Environmental Science, Qingdao National Laboratory for Marine Science and Technology, Qingdao, China; ^4^Center for Ocean Mega-Science, Chinese Academy of Sciences, Qingdao, China; ^5^College of Marine Science, University of Chinese Academy of Sciences, Beijing, China; ^6^Department of Molecular Biology and Biochemistry, Simon Fraser University, Burnaby, BC, Canada

**Keywords:** *Chaetoceros* species, chloroplast genome, comparative genomics, variation hotspots, divergence time

## Abstract

*Chaetoceros* is a species-rich diatom genus with broad distribution and plays an important role in global carbon cycle and aquatic ecosystems. However, genomic information of *Chaetoceros* species is limited, hindering advanced researches on *Chaetoceros* biodiversity and their differential impact on ecology. In this study, we constructed full-length chloroplast genomes (cpDNAs) for seven *Chaetoceros* species, including *C. costatus*, *C. curvisetus*, *C. laevisporus*, *C. muelleri*, *C. pseudo-curvisetus*, *C. socialis*, and *C. tenuissimus*. All of these cpDNAs displayed a typical quadripartite structure with conserved genome arrangement and specific divergence. The sizes of these cpDNAs were similar, ranging from 116,421 to 119,034 bp in size, and these cpDNAs also displayed similar GC content, ranging from 30.26 to 32.10%. Despite extensive synteny conservation, discrete regions showed high variations. Divergence time estimation revealed that the common ancestor of *Chaetoceros* species, which formed a monophyletic clade at approximately 58 million years ago (Mya), split from *Acanthoceras zachariasii* at about 70 Mya. The availability of cpDNAs of multiple *Chaetoceros* species provided valuable reference sequences for studying evolutionary relationship among *Chaetoceros* species, as well as between *Chaetoceros* species and other diatom species.

## Introduction

Diatoms (Bacillariophyta) are one of the most diverse lineages of phytoplankton on earth, with approximately 200,000 species (Mann and Droop, 1996; Malviya et al., 2016). As primary producers, they play an important role in aquatic food webs and in biogeochemical cycles (Smetacek, 1998; Armbrust, 2009).

*Chaetoceros* Ehrenberg is a species-rich genus of the class Mediophyceae (Rine, 1988; Kooistra et al., 2010; Malviya et al., 2016; Gaonkar et al., 2018; De Luca et al., 2019a) with 232 taxonomically accepted species (accessed on June 2021) (Guiry and Guiry, 2021). As one of the largest genera of planktonic diatom, *Chaetoceros* plays an important role in global carbon cycle and aquatic ecosystems (Nelson et al., 1995). *Chaetoceros* species play an important role in ecological systems as an important component of natural food webs. As such, some *Chaetoceros* species are often cultivated to serve as feed for aquaculture of shellfish, shrimp, and fish because of its high nutrition content (Göksan et al., 2003; Liang et al., 2020). Additionally, some *Chaetoceros* species have been used as biological indicators for studying marine environmental change (Wang et al., 2010). Furthermore, many *Chaetoceros* species have been applied to remove certain antibiotics from wastewater (Mojiri et al., 2021), and *C. muelleri* has been exploited as a renewable precursor to liquid fuels or as a lipid source because of its high growth rate, tolerance to a broad range of temperatures, and specific conductance and large quantity of intracellular lipid (McGinnis et al., 1997; López-Elías et al., 2005; Yin and Hu, 2021).

Nevertheless, many *Chaetoceros* species can also pose negative impact on environment by inducing harmful algal blooms (HABs) under certain circumstances. HABs caused by various *Chaetoceros* species have been reported in many countries including Japan (Oyama et al., 2008; Tomaru et al., 2011; Tomaru et al., 2017), Spain (Trigueros et al., 2002), the United States (Montresor et al., 2013), India (Begum et al., 2015), and China (Lv et al., 1993; Han et al., 2004; Liu et al., 2006; Wang et al., 2010). Some *Chaetoceros* species can also negatively impact aquaculture and fisheries (Albright et al., 1993; Treasurer et al., 2003; Begum et al., 2015). For example, *Chaetoceros densus* has been found to impact the *Porphyra yezoensis* cultures in Japan (Oyama et al., 2008), and *C. convolutus* and *C. concavicornis* can cause fish mortality by anchoring the setae to the sensitive gill tissue (Albright et al., 1993; Treasurer et al., 2003; Wang et al., 2008).

*Chaetoceros* species are generally easily recognized among diatom species by the chain-forming cells that are separated by apertures, and the long setae protruding from each of the four corners of the cells. A small minority are solitary in their growth form (Round et al., 1990; Li et al., 2017). Nevertheless, *Chaetoceros* species could not be accurately characterized due primarily to their high morphological similarities. New species (Marino et al., 1991; Rines et al., 2010; Yang et al., 2015) and cryptic species (Chamnansinp et al., 2013, 2015; Balzano et al., 2017; Li et al., 2017) are being uncovered, suggesting that a considerable part of the diversity in the *Chaetoceros* is still to be revealed.

Molecular markers have been applied to distinguish and describe taxa, including species in Chaetocerotaceae. Gaonkar et al. (2018) used the full-length or partial 18S rDNA and partial 28S rDNA as molecular markers to enable phylogenetic inference of species in Chaetocerotaceae, but often could not be used to accurately distinguish different species of a same genus. Although concatenated alignment of multiple molecular markers such as 18S rDNA, partial 28S rDNA, *rbcL*, *psbA*, and partial COI could enhance resolution capability (De Luca et al., 2019b), molecular markers with even higher resolution are urgently needed to enrich public databases for research on biodiversity and evolution.

Chloroplast genomes (cpDNAs) have been used as “super-barcode” for comparative genomics analysis (Fu et al., 2019; Ji et al., 2019). cpDNA has a typical quadripartite structure consisting of one large single copy region (LSC), one small single copy region (SSC), and a pair of inverted repeats (IRs) (Bendich, 2004; Daniell et al., 2016). The complete cpDNAs have been shown to be valuable in inferring evolutionary relationships as an accessible genetic resource (Yu et al., 2018). With the recent development of DNA sequencing technologies, a growing number of cpDNAs of species in Bacillariophyta have been fully constructed (Yu et al., 2018; Yao et al., 2021; Zhang et al., 2021). Comparative analysis of cpDNAs can help us understand the complex evolutionary relationships of algal species. In addition, comparative analysis of cpDNAs can also be applied as an effective method to develop high-resolution molecular markers (Ji et al., 2019; Song et al., 2020).

Fossil evidence suggests that diatoms originated in the late Jurassic period (Finkel et al., 2005; Lewitus et al., 2018). Chaetocerotaceae spores sink out of the water column and can remain dormant in the sediment for prolonged periods, so species in this family exhibit extensive fossil records (Suto, 2006). However, the biodiversity of existent *Chaetoceros* species has not been adequately explored. Comparative analysis of fossil records and cpDNAs of *Chaetoceros* species may provide valuable insight into the understanding of origin and evolution of *Chaetoceros* species.

By now, cpDNAs of only two *Chaetoceros* species (i.e., *C. muelleri* and *C. simplex*) have been constructed (Sabir et al., 2014; Li and Deng, 2021). In this study, we constructed full-length cpDNAs for seven *Chaetoceros* species, including *C. costatus*, *C. curvisetus*, *C. laevisporus*, *C. muelleri*, *C. pseudo-curvisetus*, *C. socialis*, and *C. tenuissimus*, all of which were isolated from coastal waters in China. Comparative analysis of these cpDNAs revealed extensive gene and synteny conservation, as well as the identification of several variation hotspots. We also explored phylogenetic analysis and divergence time for *Chaetoceros* species and other species in the diatom.

## Materials and Methods

### Strain Isolation and Whole Genome Sequencing

**FIGURE 1:**
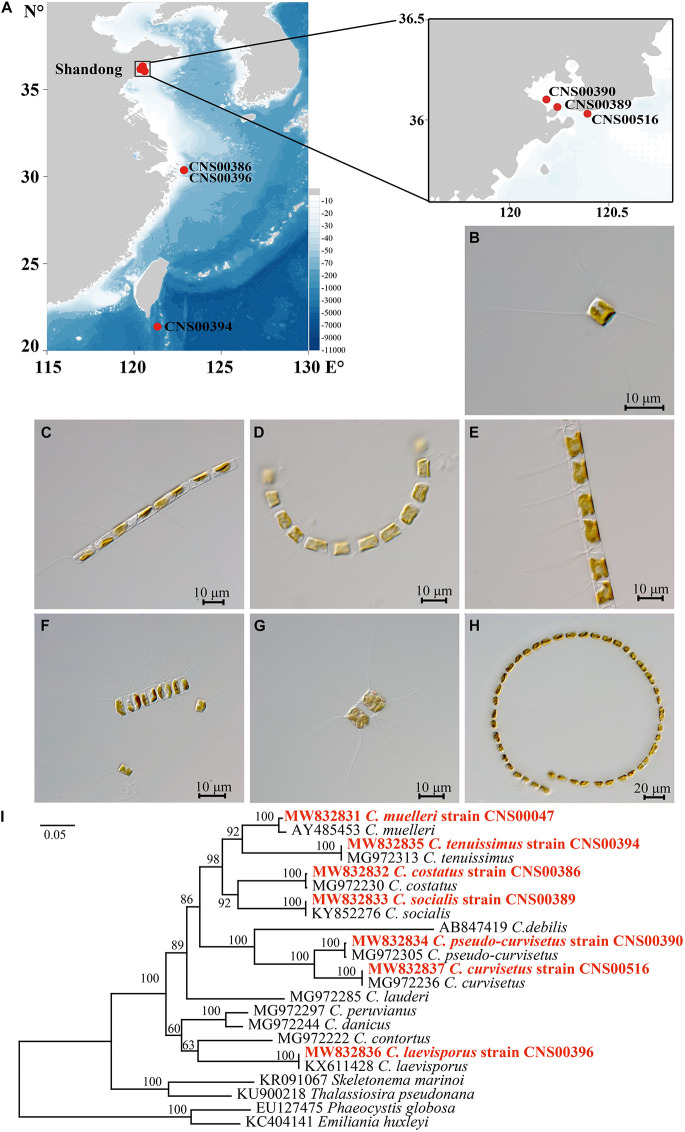
**(A)** Sampling locations of *Chaetoceros* species. **(B–H)** The micrographs of *Chaetoceros* species. **(B)** *C. muelleri* (strain CNS00047). **(C)** *C. costatus* (strain CNS00386). **(D)** *C. socialis* (strain CNS00389). **(E)** *C. pseudo-curvisetus* (strain CNS00390). **(F)** *C. tenuissimus* (strain CNS00394). **(G)** *C. laevisporus* (strain CNS00396). **(H)** *C. curvisetus* (strain CNS00516). **(I)** The phylogenetic analysis of *Chaetoceros* species and outgroups (*Phaeocystis globosa* and *Emiliania huxleyi*) using full length of 18S rDNA gene.

Seven candidate *Chaetoceros* species studied in this project were isolated from water samples collected during multiple expeditions in Chinese coastal waters, among which CNS00389, CNS00390, and CNS00516 were isolated from the Jiaozhou Bay (July and August, 2019) on the research vehicle “Chuangxin,” CNS00386 and CNS00396 were isolated from the Changjiang Estuary (July, 2019) on the research vehicle “Zheyu 2,” and CNS00394 was isolated from the East China Sea (May, 2019) on the research vehicle “Xiang Yang Hong 18” (Figure 1A and Supplementary Table 1). The *Chaetoceros* cells were individually isolated using a micropipette, followed by multiple washes before transferring each single cell to 24-well culture dishes for growth and characterization. These *Chaetoceros* strains were cultured in L1 medium with 1 ‰ volume fraction Na_2_SiO_3_ with H_2_O added (Guillard and Hargreaves, 1994). The culture temperature was set at 19 ± 1°C, and the illumination intensity was from 2000Lx to 3000Lx at the photoperiod of 12 h light-12 h dark.

Identification of the cultured *Chaetoceros* strains was done according to both microscopic morphological characters and phylogenetic analysis using universal markers, including full-length 18S rDNA, *rbcL*, and 28S rDNA D1-D3. The morphological features of the *Chaetoceros* species were observed using ZEISS Axio Imager 2 (ZEISS, Germany). Molecular marker sequences were assembled using Illumina reads with SPAdes (Bankevich et al., 2012) and GetOrganelle (Jin et al., 2020), with publicly available molecular marker sequences of *Chaetoceros* species as reference sequences. The assembled sequences were validated by the following steps. (1) Reads were aligned to the assembled sequences using BWA (0.7.17) (Li and Durbin, 2009). (2) Alignment results were extracted using SAMtools (1.10) (Li et al., 2009). (3) Resulting alignments were inspected for validation and error correction using IGV (Thorvaldsdottir et al., 2013). Phylogenetic trees based on molecular markers were constructed using MEGAX (Kumar et al., 2018). Phylogenetic relationships were inferred using the Maximum Likelihood (ML) (Tamura et al., 2004). The percentage of replicate trees in which the associated taxa clustered together in the bootstrap test (1000 replicates) was shown next to the branches (Felsenstein, 1985).

For DNA library preparation for whole genome sequencing, *Chaetoceros* cells were collected by centrifugation, and algae mud samples were stored in liquid nitrogen for subsequent DNA extraction. Total DNA was extracted for each sample by using DNAsecure Plant Kit (Tiangen Biotech, Beijing, China). The integrity and purity of DNA were examined by 1% agarose gel electrophoresis and DNA concentration was accurately quantified by Qubit 2.0 Flurometer (Life Technologies, CA, United States). DNA libraries were prepared using NEB Next^TM^ Ultra^®^ DNA Library Prep Kit for Illumina (NEB, United States). PCR products were purified using AMPure XP system (Beckman Coulter, Beverly, MA, United States), libraries were analyzed for size distribution using NGS3K/Caliper and quantified using real-time PCR (Qubit^®^3.0 Flurometer, Invitrogen, United States). Qualified libraries were sequenced using NovaSeq PE150 (Illumina, San Diego, CA, United States) at Novogene (Beijing, China).

### Chloroplast Genome Assembly and Annotation

We obtained an average of 5.85 Gb of Illumina paired-end clean sequencing data from genomic DNA of seven *Chaetoceros* strains. An average of 19,511,552 paired-end reads were retrieved from each sample, with a sequence length of 150 bp. Raw reads in FASTQ format were first processed through a series of quality control (QC) procedures to obtain clean reads, according to method described previously (Song et al., 2020). Complete cpDNAs were assembled using the GetOrganelle (Jin et al., 2020) with clean reads. Chloroplast genome sequences were verified using the same method used for verifying molecular markers described above in 2.1. Annotation of cpDNA was made using MFannot^1^ using genetic code of Bacterial, Archaeal, and Plant chloroplast. For genes whose lengths were different from expected, whose start and stop codons were non-canonical, or open reading frames (*orfs*) that did not show similarity to known genes, Open Reading Frame Finder (ORF finder)^2^ was applied to examine and edit gene models. Annotated results were further validated and formatted using NCBI’s Sequin15.10^3^. cpDNAs in the Genbank format of the cpDNAs were converted into genome maps by using Organellar Genome DRAW (OGDRAW) online software (Greiner et al., 2019).

### Phylogenetic Analysis

Phylogenetic tree based on protein-coding genes (PCGs) was constructed using extracting 95 PCGs (Supplementary Table 2) shared by published Bacillariophyta cpDNAs (Supplementary Table 3) including seven *Chaetoceros* cpDNAs constructed in this study. The amino acid sequences of each of the 95 PCGs from different diatom cpDNAs were individually aligned using MAFFT v7.310 (–auto) (Katoh and Standley, 2013). Regions that were ambiguously aligned in each alignment were deleted and all amino acid sequences were concatenated using PhyloSuite v1.2.2 (Zhang et al., 2020). Phylogenetic tree was constructed using IQ-TREE v1.6.1 with SH-aLRT support (%)/aBayes support/ultrafast bootstrap support (%) (parameters: -st AA -m TEST -bb 1000 -alrt 1000 -abayes) (Trifinopoulos et al., 2016). *Triparma laevis* (AP014625) in Ochrophyta was included as out-group taxa.

### Genome Comparison

Alignment of *Chaetoceros* cpDNAs were performed by using Mauve v2.4.0 (Darling et al., 2010) with default parameters. The cpDNAs borders were analyzed to show the IR expansions and contractions using irscope_pack.3.1 (modified from IRscope) (Amiryousefi et al., 2018).

### Identification of Variation Hotspot Regions

*Chaetoceros* cpDNAs were aligned using MAFFT v7.310 (Katoh and Standley, 2013). Nucleotide diversity (Pi), which could be used to estimate the degree of nucleotide sequence variations, which could be used as potential molecular markers, was calculated using the software DnaSP v6.12.03 (Rozas et al., 2017) and cpDNA alignment as input. The window size was set to 600 bp and the step size was 50 bp.

### Divergence Time Estimations

Divergence time estimation was performed by 95 PCGs (Supplementary Table 2) shared by the published 55 Bacillariophyta cpDNAs (Supplementary Table 3) and seven *Chaetoceros* cpDNAs constructed in this study using MCMCTree in PAML v4.8a (Yang, 2007). Branch lengths, gradient (g) and Hessian (H) were estimated using maximum likelihood estimates (MLE) and GTR + G substitution model (model = 7) with independent rates clock model (clock = 1). Three calibration points^4^ were included in this analysis (Supplementary Table 4), including the calibration point between *Ectocarpus siliculosus* and diatoms [176.0–202.0 Million years ago (Mya)], the calibration point between *Rhizosolenia setigera* and *Skeletonema pseudocostatum* (90.5–91.5 Mya), and the calibration point between *Pseudo-nitzschia multiseries* and *Fragilariopsis cylindrus* (10.0–35.3 Mya). The phylogenetic tree was displayed using FigTree v1.4.3 and visualized with 95% highest posterior density interval (HPD) for each node.

## Results

### Morphological and Molecular Identification of Seven *Chaetoceros* Species

All seven *Chaetoceros* species studied in this project formed chains in which cells were separated by apertures, with long setae protruding from each of the four corners of the cells. These *Chaetoceros* strains all displayed substantial morphological variations (Li et al., 2017; Xu et al., 2020). The strain CNS00047 was annotated as *C. muelleri* because these cells were rectangular with long setae, with valve diameter varying from 4.5 to 20.0 μm (Figure 1B), similar to previous description of *C. muelleri* (Reinke, 1984). Phylogenetics analysis of full-length 18S rDNA sequences of these candidate *Chaetoceros* species and reference sequences of known *Chaetoceros* species confirmed that the strain CNS00047 was *C. muelleri* because its 18S rDNA sequence (MW832831) clustered well with that (AY485453) of *C. muelleri* (Damste et al., 2004) with high percentage identity (PID) 99.43% (Figure 1I and Table 1). This annotation was also supported by phylogenetic analysis using another molecular marker *rbcL* (Supplementary Figure 1A and Table 1). The strain CNS00386 was annotated as *Chaetoceros costatus*, which contained a single, lobed plastid, formed straight chains (Figure 1C; Kooistra et al., 2010). The strains CNS00389 was annotated as *C. socialis*, which was fan-shaped, with one of the four setae longer than the others and the long setae of adjacent cells joining together (Figure 1D; Degerlund et al., 2012; Pelusi et al., 2019). The strain CNS00390 was annotated as *C. pseudo-curvisetus*, whose chains were curved, with a large aperture between adjacent cells, and the aperture was large in the middle and small on the sides (Figure 1E; Oku and Kamatani, 1990). The strain CNS00394 was annotated as *C. tenuissimus*, whose cells were very small, being square to rectangular, with setae being narrow, arising from the two poles of the valve at an angle of 45° to its apical axis (Figure 1F; Sar et al., 2002). The strain CNS00396 was annotated as *C. laevisporus*, whose cells contained multiple plastids, were rectangular in broad girdle view and formed straight chains (Figure 1G; Chen et al., 2019). The strain CNS00516 was annotated as *C. curvisetus*, whose chains were helical, with a large elliptical aperture between adjacent cells. Each cell contained only a single plastid, and all setae curve toward the convex side of the chain (Figure 1H). The strains CNS00386, CNS00389, CNS00390, CNS00394, CNS00396, and CNS00516 were further confirmed as *C. costatus* (Gaonkar et al., 2018), *C. socialis* (Gaonkar et al., 2017), *C. pseudo-curvisetus* (Gaonkar et al., 2018), *C. tenuissimus* (Gaonkar et al., 2018), *C. laevisporus* (Li et al., 2017), and *C. curvisetus* (Gaonkar et al., 2018), respectively, according to their molecular features (Figure 1, Supplementary Figure 1, and Table 1).

**TABLE 1:** Basic characteristics of *Chaetoceros* cpDNAs.

Species		*C. muelleri*	*C. costatus*	*C. socialis*	*C. pseudo-curvisetus*	*C. tenuissimus*	*C. laevisporus*	*C. curvisetus*	*C. muelleri*	*C. simplex*
Strains		CNS00047	CNS00386	CNS00389	CNS00390	CNS00394	CNS00396	CNS00516	–	–
Reference		This study	This study	This study	This study	This study	This study	This study	Li and Deng, 2021	Sabir et al., 2014
Access No.		MW845774	MW845775	MW845776	MW845777	MW845778	MW845779	MW845780	NC_053621	NC_025310
18S rDNA annotation		*C. muelleri* (AY485453, 99.43%)	*C. costatus* (MG972230, 99.82%)	*C. socialis* (KY852276, 100.00%)	*C. pseudo-curvisetus* (MG972305, 99.82%)	*C. tenuissimus* (MG972313, 99.94%)	*C. laevisporus* (KY611428, 100.00%)	*C. curvisetus* (MG972236, 100.00%)	–	–
*rbcL* annotation		*C. muelleri* (HQ912422 97.22%)	*C. costatus* (MK642509 100.00%)	*C. socialis* (MK642547, 100%)	*C. pseudo-curvisetus* (MK642540, 99.82%)	*C. tenuissimus* (MK642556, 99.47%)	*C. laevisporus* (MK642524, 97.26%)	*C. curvisetus* (MK642514, 99.92%)	–	–
Size (bp)		116,421	116,845	117,717	118,127	116,523	119,034	118,222	116,284	116,459
IR length (bp)		7576	6995	7257	7538	7411	8039	7580	7515	7403
LSC length (bp)		61,902	63,178	63,586	63,350	62,181	63,365	63,277	61,946	62,136
SSC length (bp)		39,367	39,677	39,617	39,701	39,520	39,591	39,785	39,308	39,517
GC content		30.80%	32.10%	31.27%	31.55%	32.06%	30.26%	31.80%	30.87%	32.07%

**Total number of genes**		169	166	168	168	169	171	168	168	169
PCGs		131	128	131	131	131	133	131	131	131
tRNA		30	30	30	30	30	30	30	30	30
rRNA		6	6	6	6	6	6	6	6	6
ncRNA		1	1	0	0	1	1	0	0	1
tmRNA		1	1	1	1	1	1	1	1	1

**Intergenic regions (bp)**	Total	14,783	15,512	15,935	16,358	14,489	16,621	16,499	14,425	14,823
	Max	350	486	1008	453	321	464	425	381	319
	Min	2	1	1	1	1	1	1	2	1
	Median	63	65	65	65	63	66	65	65	61

### Construction and Comparative Analysis of Chloroplast Genomes

**FIGURE 2:**
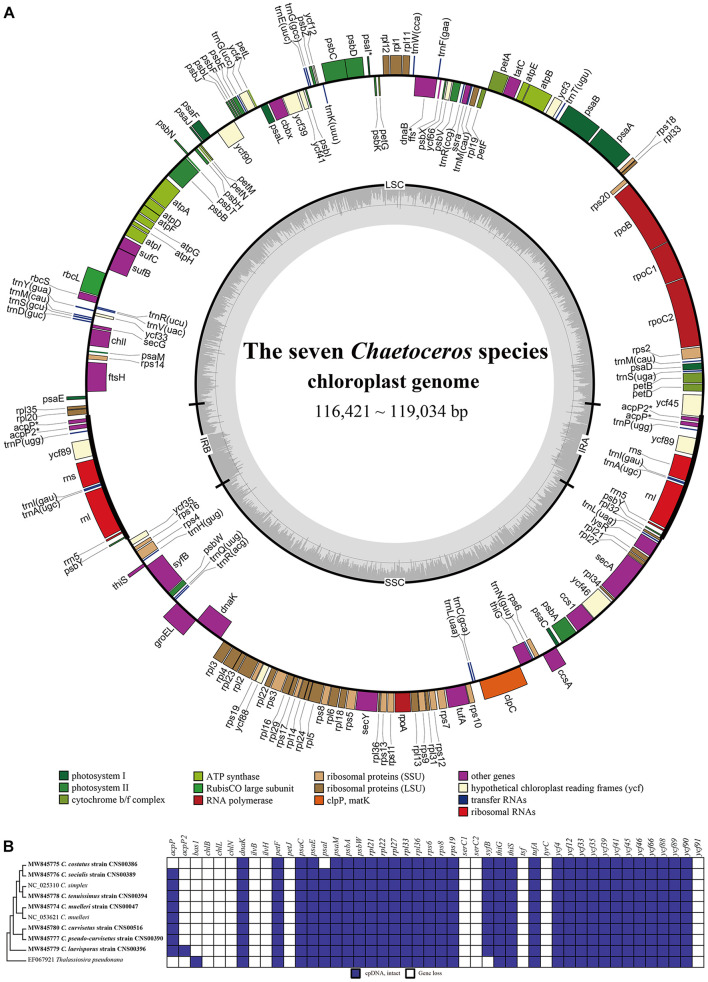
Gene structural and evolutionary patterns of genes of the *Chaetoceros* cpDNAs. **(A)** Map of cpDNAs of the seven *Chaetoceros*. Dashed area in the inner circle indicates the GC content of the cpDNA. Genes belonging to different functional groups are color-coded as indicated by icons on the lower. The asterisk represents differences between the seven *Chaetoceros*. **(B)** Evolutionary patterns of loss, and gain of genes in *Chaetoceros* cpDNAs. The matrix shows 46 genes variably present among the sequenced genomes. Taxa in boldface identify genomes sequenced for this study. Phylogenetic relationships based on concatenated amino acid sequences of protein-coding genes using Maximum likelihood (ML) methods. *T. pseudonana* was used as out-group taxa.

We constructed full-length cpDNAs for seven *Chaetoceros* species, among which cpDNAs of six *Chaetoceros* species (*C. costatus*, *C. curvisetus*, *C. laevisporus*, *C. pseudo-curvisetus*, *C. socialis*, and *C. tenuissimus*) were constructed for the first time. Together with two cpDNAs of *C. muelleri* and *C. simplex* that have been previously published (Sabir et al., 2014; Li and Deng, 2021), altogether nine cpDNAs representing eight *Chaetoceros* species (Table 1) were analyzed in this project. The sizes of these nine *Chaetoceros* cpDNAs were rather similar, ranging from 116,284 bp (*C. muelleri*; NC_053621) to 119,034 bp (*C. laevisporus*; MW845779) (Figure 2A, Supplementary Figure 2, and Table 1). The GC contents of these cpDNAs were also similar (30.26–32.10%). These *Chaetoceros* cpDNAs all formed typical quadripartite structure with two inverted repeats regions (IRa, IRb), a large single copy (LSC) region, and a small single copy (SSC) region (Figure 2 and Supplementary Figure 2). The lengths of LSC regions of these cpDNAs were similar (ranging from 61,902 to 63,586 bp), so were their SSC regions (ranging from 39,367 to 39,785 bp). In contrast, the lengths of IR regions showed larger variations among these cpDNAs, with the shortest being 6995 bp (*C. costatus*), while the longest being 8039 bp (*C. laevisporus*) (Table 1).

Annotation of these cpDNAs revealed that the cpDNAs of five species, including *C. curvisetus*, *C. muelleri*, *C. pseudo-curvisetus*, *C. socialis*, and *C. tenuissimus*, each contained 131 PCGs. In contrast, the cpDNAs of *C. costatus* and *C. laevisporus* contained different numbers of PCGs, and *C. costatus* and *C. laevisporus* contained 128 and 133 genes, respectively (Figure 2A, Supplementary Figure 2, and Table 1). All *Chaetoceros* cpDNAs contained 30 tRNA and six non-coding rRNA genes (*rns*, *rnl*, and *rrn*5 in IRs) (Figure 2A, Supplementary Figure 2, and Table 1). No introns were found in any of the cpDNAs of these seven *Chaetoceros* species, which was consistent to previous findings that no introns were identified in cpDNAs of *C. simplex* (NC_025310) and *C. muelleri* (NC_053621).

A comparison of 46 genes variably present among the diatom cpDNAs between these nine *Chaetoceros* strains and *Thalassiosira pseudonana* revealed many instances of gene gains and losses (Figure 2B). Except for *acpP*, *acpP2*, and *psaI*, the presence or absence of genes in *Chaetoceros* was generally consistent. These events included peroxiredoxin gene (*bas1*), three genes encoding subunits of protochlorophyllide reductase (*chlB*/*L/N*), the large and small subunits of acetolactate synthase (*ilvB*/*H*), cytochrome C6 gene (*petJ*), two putative serine recombinase genes (*serC1* and *serC2*), putative tyrosine recombinase gene (*tyrC*), florigen genes (*tsf*), and hypothetical protein *ycf91* (Figure 2B).

These cpDNAs were rather compact, with small intergenic regions, and the median lengths of intergenic regions ranged from 63 to 66 bp. The total lengths of intergenic regions of these nine cpDNAs were similar, ranging from 14,425 bp (12.4% of the total cpDNA of *C. mueller*i; NC_053621) to 16,621 bp (14.0% of the total cpDNA of *C. laevisporus*).

### Phylogenetic Analysis of *Chaetoceros* Chloroplast Genomes

**FIGURE 3:**
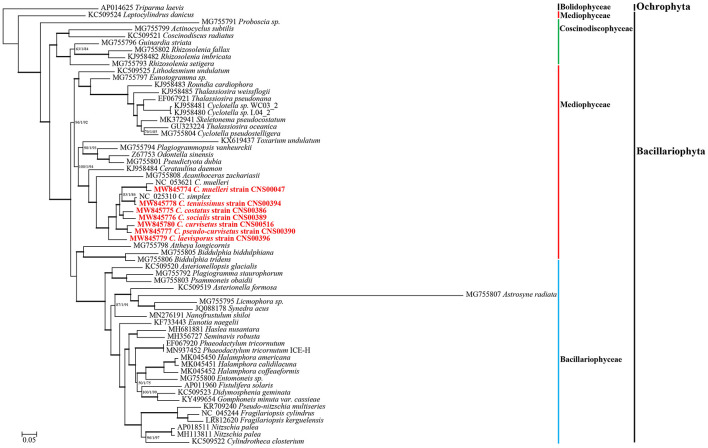
The phylogenetic tree based on concatenated amino acid sequences of 95 shared protein-coding genes using Maximum likelihood (ML) methods. *Triparma laevis* was used as out-group taxa. Numbers on the branches represent SH-aLRT support (%), aBayes support, and ultrafast bootstrap support (%), respectively. Support values are shown only for branches that did not come with high confidence level (100/1/100). Thick branches indicate high confidence level (100/1/100).

To explore the evolutionary relationship between *Chaetoceros* species and other diatom species, we constructed a phylogenetic tree using 95 PCGs (Supplementary Table 2) that were shared by 62 cpDNAs constructed for Bacillariophyta species (including six *sp.*). The cpDNA of *T. laevis* (AP014625), which belonged to the class Bolidophyceae in phylum Ochrophyta, was included as an out-group taxon (Figure 3). All Bacillariophyta species were clustered into three major clades in the phylogenetic tree, corresponding to three classes including Mediophyceae, Bacillariophyceae, and Coscinodiscophyceae, respectively (Figure 3). As expected, all nine cpDNAs of the *Chaetoceros* species clustered into a single clade. In particular, the cpDNA of *C. muelleri* (MW845774) and that of *C. muelleri* (NC_053621) clustered together, and the cpDNA of *C. tenuissimus* (MW845778) clustered closely with that of *C. simplex* (NC_025310) (Gaonkar et al., 2018; Figure 3). Additionally, cpDNAs of *C. curvisetus* and *C. pseudo-curvisetus* clustered closely, which was consistent with previous report (Gaonkar et al., 2018). In contrast, cpDNAs of *C. laevisporus* formed an independent clade (Li et al., 2017). The *Chaetoceros* clade clustered closely with *Acanthoceras zachariasii* (Chaetocerotaceae), which was consistent with previous study (Yu et al., 2018; Li and Deng, 2021).

### Synteny Analysis of *Chaetoceros* Chloroplast Genomes

**FIGURE 4:**
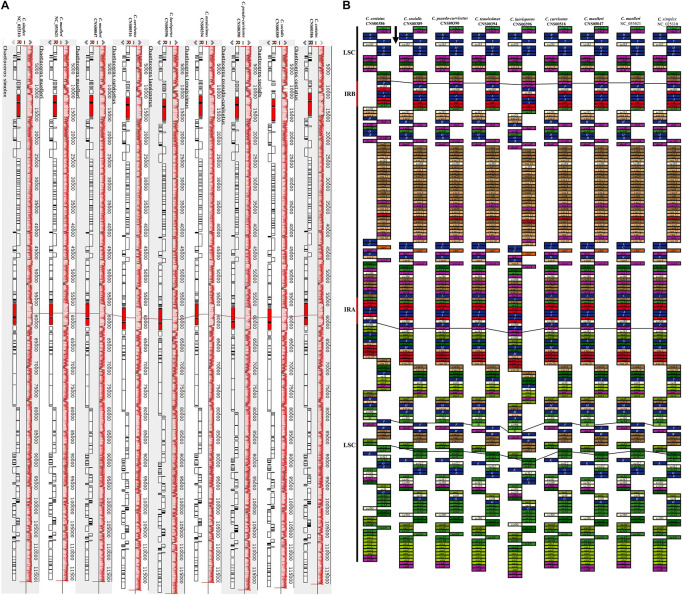
Synteny relationships and gene arrangements of *Chaetoceros.* **(A)** Synteny relationships among *Chaetoceros* cpDNAs. **(B)** Gene arrangements of *Chaetoceros* species. Blocks with the same color represent the same type of genes. The black arrow indicates transcription direction.

Comparative analysis of *Chaetoceros* cpDNAs revealed near perfect synteny among nine cpDNAs of eight *Chaetoceros* species (Figure 4A). All genes in the nine cpDNAs exhibited nearly identical gene order (Figure 4B), with only four minor differences identified. First, while a single gene *acpP* (234–246 bp) was found between *rpl20* and *trnP* in the IRb in the cpDNAs of *C. curvisetus*, *C. muelleri* (both MW845774 and NC_053621), *C. pseudo-curvisetus*, *C. simplex*, *C. socialis*, and *C. tenuissimus*, two genes *acpP* (240 bp) and *acpP2* (267 bp) were found in the corresponding region in the cpDNA of *C. laevisporus*, and no genes were found in the same region in the cpDNA of *C. costatus*. Second, the same difference was also found in the IRa. Third, while a single protein-associated ncRNA gene *ffs* (109 bp) was found between the two genes *psbX* and *trnF* in the cpDNAs of *C. costatus*, *C. laevisporus*, *C. muelleri* (MW845774), *C. simplex*, and *C. tenuissimus*, it was not found in the corresponding region in the cpDNAs of *C. curvisetus*, *C. muelleri* (NC_053621), *C. pseudo-curvisetus*, and *C. socialis*. Notably, cpDNAs of two *C. muelleri* strains were different at this site as well, while *ffs* was found in *C. muelleri* (MW845774), it was not found in the cpDNA of *C. muelleri* (NC_053621), suggesting that this site was highly polymorphic. Lastly, the gene *psaI* was found in all cpDNAs studied in this project, except the *C. costatus* cpDNA.

### Expansion and Contraction of Inverted Regions

**FIGURE 5:**
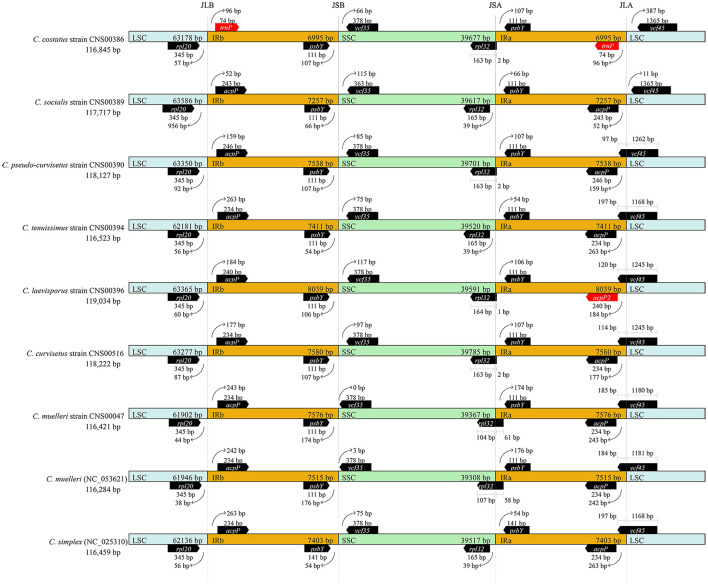
Comparison of the junctions between the LSC, SSC, and IR regions among *Chaetoceros* cpDNAs.

Comparative analysis of the nine *Chaetoceros* cpDNAs revealed that the IR regions were generally similar, but with important differences (Figure 5). In particular, the IRa/LSC and IRb/SSC boundaries were different among these cpDNAs. Except for the *C. costatus* cpDNA, the distances between *rpl20* and the LSC/IRb boundaries ranged from 38 to 956 bp, while the distances between *acpP* and the LSC/IRb boundaries ranged from 52 to 263 bp. The distances between *psbY* and the SSC/IRb boundaries ranged from 54 to 176 bp, while the distances between *ycf35* and the SSC/IRb boundaries ranged from 0 to 117 bp. Except for the *C. simplex* cpDNA, the *C. socialis* cpDNA, and the *C. tenuissimus* cpDNA, *rpl32* was located at the SSC/IRa boundaries. Except for the *C. costatus* cpDNA and the *C. socialis* cpDNA, *ycf45* was located at the SSC/IRa boundaries (Figure 5). Because of the loss of the *acpP* gene in the *C. costatus* cpDNA, the boundary between the LSC/IRb shifted, causing a contraction of both IRa and IRb regions. The IRa and IRb of the *C. costatus* cpDNA each contained eight genes (*trn*P-UGG, *ycf89*, *rns*, *trn*I-GAU, *trn*A-UGC, *rnl*, *rrn5*, and *psbY*), compared to nine genes in the cpDNAs of *C. curvisetus*, *C. muelleri*, *C. pseudo-curvisetus*, *C. simplex*, *C. socialis*, and *C. tenuissimus*. In contrast, the IRa and IRb of the *C. laevisporus* cpDNA each contained 10 genes.

### Variation Hotspots in the Chloroplast Genomes of *Chaetoceros* Species

**FIGURE 6:**
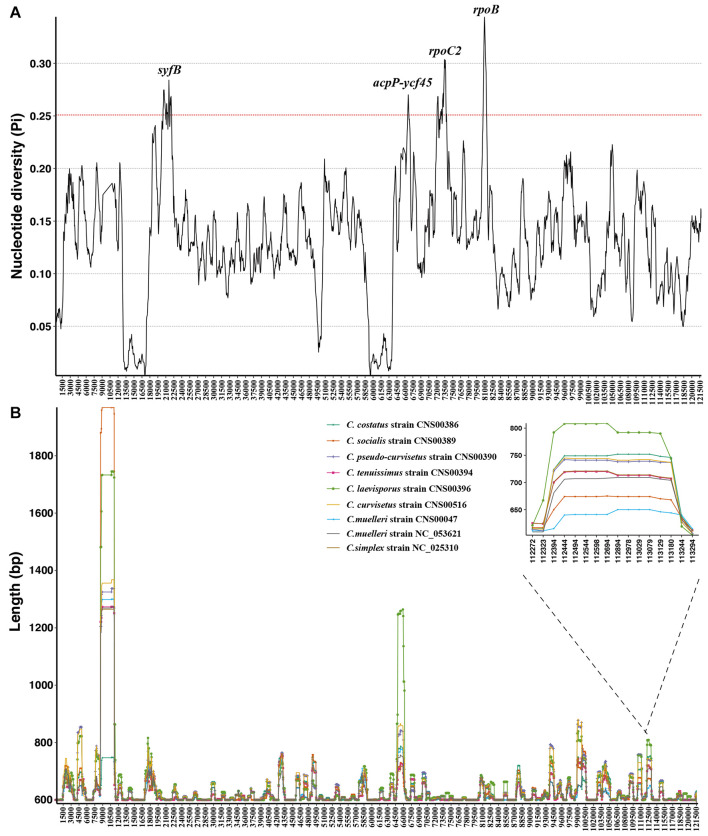
Sliding window analysis of aligned whole cpDNAs of the *Chaetoceros*. Window length, 600 bp; step size, 50 bp. **(A)** Based on nucleotide diversity values and **(B)** the distribution of indel.

Although these nine *Chaetoceros* cpDNAs showed generally high collinearity, local regions of these cpDNA sequences showed substantial variations at the DNA level. To quantify sequence divergence in these *Chaetoceros* cpDNAs, we calculated and compared the nucleotide diversity (Pi) values of the *Chaetoceros* cpDNAs with a window size of 600 bp and a step size of 50 bp. Pi values ranged from 0.0031 to 0.3442. In this analysis, 62 windows were found to have high nucleotide diversity, with Pi greater than 0.25. The main regions contained *syf*B, *rop*C2, *rpo*B genes, and *acpP*-*ycf*45 (Figure 6A). Based on the total number of single nucleotide variations (SNVs) and gaps in each window and the ability to distinguish different *Chaetoceros* species, we identified a hotspots region with 354 SNVs and no gaps (position: 19,025–19,624 bp in *C. muelleri* cpDNA), which contained a gene *syfB* (Figure 6B).

To evaluate the resolution power of the hotspots region as a molecular marker, we carried out phylogenetic analysis using it and the result showed that the hotspots region could be used as molecular markers to distinguish different *Chaetoceros* species (Supplementary Figures 3A, 4A).

Furthermore, based on the sliding window analysis, we also showed distribution of variations of the *Chaetoceros* cpDNAs. We calculated the actual sequence length of each *Chaetoceros* cpDNA in each window. We identified a region with high presence and absence variations (ranging from 806 to 961 bp), which corresponded to the region spanning 106,895–107,700 bp in the *C. muelleri* cpDNA. Phylogenetic analysis of this region showed that it represented a mutation hotspot, which could be used as a potential molecular marker to distinguish different *Chaetoceros* species (Supplementary Figures 3B, 4B).

### Divergence Time Estimation Based on Protein-Coding Genes of Chloroplast Genomes

**FIGURE 7:**
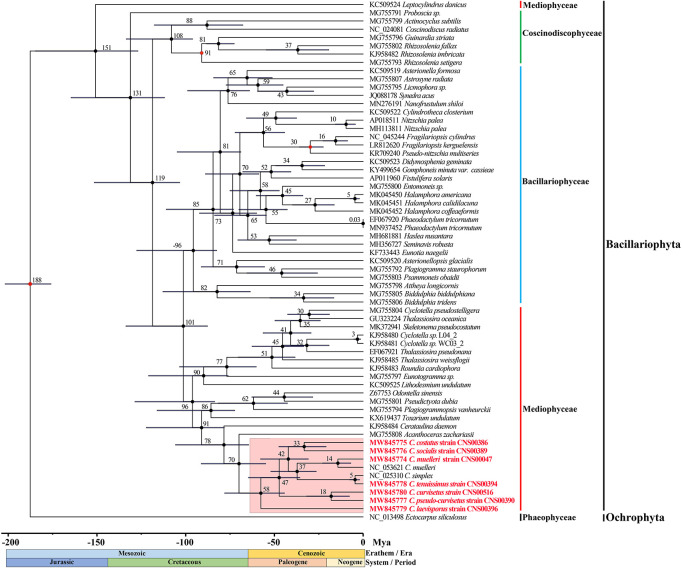
Time-calibrated phylogeny of 62 species based on 95 shared PCGs in the diatoms and outgroup (*Ectocarpus siliculosus*). The red dots represent calibration point and the 95% highest posterior density interval for node ages are shown with translucent black bars.

Divergence time estimation of the *Chaetoceros* species was achieved by analyzing DNA sequences of 95 PCGs shared by 62 cpDNAs (Figure 7). The branching of the class Coscinodiscophyceae was estimated to have occurred 131 Million years ago (Mya). The two classes Mediophyceae and Bacillariophyceae were estimated to have separated from their common ancestor 101 Mya. Furthermore, divergence time estimation revealed that the common ancestor of *Chaetoceros* species, which formed a monophyletic clade at approximately 58 Mya, split from *A. zachariasii* (Chaetocerotaceae) at about 70 Mya. Among the *Chaetoceros* species, the age estimate for *C. laevisporus* was 58 Mya. The divergence time between *C. costatus* and *C. socialis* was inferred to have occurred at 33 Mya. The branching of *C. muelleri* was estimated to have occurred 37 Mya and diverged into different strains at 14 Mya. And the divergence time between *C. curvisetus* and *C. pseudo-curvisetus* was inferred to have occurred at about 18 Mya. While the divergence time between *C. simplex* and *C. tenuissimus* was estimated at 5 Mya. Taken together, the majority of these *Chaetoceros* species arose within 50 Mya.

## Discussion

In this study, cpDNAs of seven *Chaetoceros* species were constructed for the first time, increased the number of *Chaetoceros* cpDNAs from two to nine. This research represented a major step toward in-depth understanding of biodiversity, ecology, and speciation of *Chaetoceros*, which is a species-rich, widespread and abundant diatom genus and plays an important role in global carbon cycle and aquatic ecosystems (Nelson et al., 1995; De Luca et al., 2019a).

General features of cpDNAs of *Chaetoceros* species constructed in this study were comparable to that of cpDNAs of other diatom species, whose cpDNAs vary widely in size, ranging from 111,539 bp in *Pseudo-nitzschia multiserie*s (Cao et al., 2016) to 201,816 bp in *Plagiogramma staurophorum* (Yu et al., 2018). The sizes of cpDNAs of *Chaetoceros* species were generally similar, ranging from 116,421 bp to 119,034 bp. IR contraction and expansion, gene loss and gain, presence and absence of introns, and the variation of intergenic regions are the major factors contributing to variations in the sizes of cpDNAs (Zhu et al., 2016). Comparative analysis revealed that the variation of *Chaetoceros* cpDNA lengths was mainly driven by the variations of IR lengths and intergenic regions. The IR regions of the *Chaetoceros* cpDNA varied from 6995 bp in *C. costatus* to 8039 bp in *C. laevisporus* (Table 1). Moreover, the total length of intergenic regions of the *Chaetoceros* cpDNA ranged from 14,489 bp in *C. tenuissimus* to 16,621 bp in *C. laevisporus*. No introns were found in any of these *Chaetoceros* cpDNAs, which was consistent to previous reports that introns are rare in diatom cpDNAs (Ruck et al., 2014).

In addition to variations in the IR and intergenic regions, multiple instances of genes were found to be variable in the *Chaetoceros* cpDNAs. Compared to these five cpDNAs with 131 PCGs, the cpDNA of *C. costatus* lost *psaI* from LSC and *acpP* from each of its two IRs. The loss of the photosynthetic gene *psaI* was an rare event but has been reported for other photosynthetic organisms including *R. imbricate* (Sabir et al., 2014) and *R. fallax* (Yu et al., 2018), both of which are species in the class Coscinodiscophyceae. The loss of *acpP* is a common event which has been reported in *Thalassiosira* species, *Cyclotella* species, and *Synedra acus* (Galachyants et al., 2011; Sabir et al., 2014; Yu et al., 2018). In contrast, the *C. laevisporus* cpDNA gained an extra *acpP2* gene in each of the two IRs, which encoded proteins with low percentage identify (34.72%), suggesting an ancient duplication event, similar to *acpP* and *acpP2* reported in the cpDNAs in the cpDNAs of *Lithodesmium undulatum*, *Asterionella formosa*, and *Eunotia naegelii* (Ruck et al., 2014).

The presence or absence of genes in *Chaetoceros* were generally consistent, suggesting that these events may have occurred in the common ancestors of *Chaetoceros* species. The synteny of complete *Chaetoceros* cpDNAs was highly conserved, which was not unexpected because a previous study found high synteny conservation between the cpDNAs of Thalassiosirales species and non-Thalassiosirales species (Sabir et al., 2014). Our analysis found that *Chaetoceros* cpDNAs contained similar numbers of PCGs and non-coding genes with only minor exceptions. The *C. costatus* cpDNA lacked *acpP* (in IR) and *psaI*, while the *C. laevisporus* cpDNA had an extra *acpP*2 gene (in IR). It is well known that cpDNA genes tend to undergo a sequential process of transfer from the chloroplast to the nucleus (Yu et al., 2018). BLASTP searches of *acpP* (77 aa) and *psaI* (36 aa) in the assembled nuclear genome of *C. costatus* (CNS00386) and identified two putative hits with PID of 54.7% and 50.7% to *acpP* in the nuclear genome assembly, respectively, one putative hit with PID of 80.6% to *psaI* in the nuclear genome. The absence of *acpP* and *psaI* from the *C. costatus* cpDNA and the presence of their potential homologs in the nuclear genome suggested that these genes could have been transferred to the host genome.

Despite high synteny of the *Chaetoceros* cpDNAs, some high variation regions were found in DNA sequences (Figure 6). Such a region (corresponding to 19,025–19,624 bp in *C. muelleri* cpDNA) with great sequence difference might be the relatively ideal marker to distinguish *Chaetoceros* species (Supplementary Figure 3A). Another region (corresponding to 106,895–107,700 bp in *C. muelleri* cpDNA) could also be applied as a molecular marker for distinguishing *Chaetoceros* species (Supplementary Figure 3B). These potential molecular markers could be valuable because even though *Chaetoceros* species are usually easily recognized to genus level for their morphological features, precise species identification can be challenging because of morphological variations (Li et al., 2017; Xu et al., 2020). Common molecular markers including full-length 18S rDNA usually do not have adequate resolution for distinguishing *Chaetoceros* species, molecular markers with higher resolution and specificity are urgently needed. Thus, these variable regions identified in this study could be applied used as potential molecular markers that have both high specificity to *Chaetoceros* species and high resolution for distinguishing closely related *Chaetoceros* species.

Based on the phylogenetic tree of species in diatoms of 95 core PCGs in cpDNAs, we found that the first event of diversification within the diatoms occurred 188 Mya (95% HPD: 175.8–201.8 Mya) (Figure 7). Previous research suggests that diatoms arose in the lower Triassic period, perhaps as early as 250 Mya according to the molecular clock estimate (Sims et al., 2006; Lewitus et al., 2018). Other studies have suggested that the first diatom lineage is likely to have evolved any time between 183 –250 Mya ago based on 18S rDNA gene (Sorhannus, 2007), which was between the Early Triassic and Early Jurassic. Furthermore, the results suggest that most diatoms occurred Paleogene period (28–66 Mya) with many *Chaetoceros* species arose within 50 Mya. The *Chaetoceros* species were closely related to the *A. zachariasii* (Chaetocerotaceae), which was consistent with previous studies (Matari and Blair, 2014; Yu et al., 2018; Li and Deng, 2021). The branching of *Chaetoceros* species was estimated to have occurred 58 Mya. However, previous studies have reported that *Chaetoceros* species was estimated to have occurred at around 90 Mya with the research based on 18S rDNA gene (Sorhannus, 2007). Among the *Chaetoceros* genus, the strains CNS00047 and NC_053621 (Li and Deng, 2021) identified as *C. muelleri* was sister clade as expect, but we also found genetic distance between the two strains (Figure 3). The branching of *C. muelleri* was estimated to have occurred 37 Mya and diverged into different strains at 14 Mya (95% HPD: 6.9–22.0 Mya) (Figure 7), suggesting that these two *C. muelleri* strains could represent two distinct *Chaetoceros* species. *Chaetoceros simplex* diverged from *C. tenuissimus* approximately 5 Mya (95% HPD: 1.9–7.6 Mya). This study provided the divergence time among the *Chaetoceros* species based on the cpDNAs for the first time.

## Conclusion

In this study, we successfully constructed the full-length cpDNAs for seven *Chaetoceros* species. The *Chaetoceros* cpDNAs ranged from 116,421 to 119,034 bp in size and displayed similar GC content of 30.26–32.10%. Comparative analysis of these cpDNAs revealed extensive gene and synteny conservation, as well as the presence of hotspot regions with high variations. Moreover, our study explored phylogenetic and divergence times for *Chaetoceros* species and other species in the diatom.

## Data Availability Statement

The original contributions presented in the study are publicly available. These data can be found here: https://www.ncbi.nlm.nih.gov/sra/PRJNA745567 and https://www.ncbi.nlm.nih.gov/nuccore/MW845774; https://www.ncbi.nlm.nih.gov/nuccore/MW845775; https://www.ncbi.nlm.nih.gov/nuccore/MW845776; https://www.ncbi.nlm.nih.gov/nuccore/MW845777; https://www.ncbi.nlm.nih.gov/nuccore/MW845778; https://www.ncbi.nlm.nih.gov/nuccore/MW845779; https://www.ncbi.nlm.nih.gov/nuccore/MW845780; https://www.ncbi.nlm.nih.gov/nuccore/MW832831; https://www.ncbi.nlm.nih.gov/nuccore/MW832832; https://www.ncbi.nlm.nih.gov/nuccore/MW832833; https://www.ncbi.nlm.nih.gov/nuccore/MW832834; https://www.ncbi.nlm.nih.gov/nuccore/MW832835; https://www.ncbi.nlm.nih.gov/nuccore/MW832836; https://www.ncbi.nlm.nih.gov/nuccore/MW832837; https://www.ncbi.nlm.nih.gov/nuccore/MZ267682; https://www.ncbi.nlm.nih.gov/nuccore/MZ267683; https://www.ncbi.nlm.nih.gov/nuccore/MZ267684; https://www.ncbi.nlm.nih.gov/nuccore/MZ267685; https://www.ncbi.nlm.nih.gov/nuccore/MZ267686; https://www.ncbi.nlm.nih.gov/nuccore/MZ267687.

## Author Contributions

NC conceived of the project and revised the manuscript. ZC carried out strain selection, cultivation, DNA preparation, and organized genome sequencing. QX carried out genome assembly, annotation, quality control, and comparative analysis of cpDNAs. QX and ZC wrote the manuscript. All authors read and approved the final version of the manuscript.

## Conflict of Interest

The authors declare that the research was conducted in the absence of any commercial or financial relationships that could be construed as a potential conflict of interest.

## Publisher’s Note

All claims expressed in this article are solely those of the authors and do not necessarily represent those of their affiliated organizations, or those of the publisher, the editors and the reviewers. Any product that may be evaluated in this article, or claim that may be made by its manufacturer, is not guaranteed or endorsed by the publisher.

## Footnotes

1
https://github.com/BFL-lab/Mfannot

2
https://www.ncbi.nlm.nih.gov/orffinder

3
https://www.ncbi.nlm.nih.gov/projects/Sequin/

4
http://www.timetree.org/

## Supplementary Material

Supplementary Figure 1 The phylogenetic analysis of *Chaetoceros* species using full length of **(A)** *rbcL* gene and **(B)** partial 28S rDNA.

Supplementary Figure 2 Gene structural map of the seven *Chaetoceros* cpDNAs. **(A)** *C. muelleri* (strain CNS00047), **(B)** *C. costatus* (strain CNS00386), **(C)** *C. socialis* (strain CNS00389), **(D)** *C. pseudo-curvisetus* (strain CNS00390), **(E)** *C. tenuissimus* (strain CNS00394), **(F)** *C. laevisporus* (strain CNS00396), and **(G)** *C. curvisetus* (strain CNS00516).

Supplementary Figure 3 Phylogenetic trees constructed using different hotspots regions. **(A)** Position: 19,025–19,624 bp in *C. muelleri* cpDNA. **(B)** 106,895–107,700 bp in *C. muelleri* cpDNA.

Supplementary Figure 4 **(A,B)** The DNA alignment information of hotspots region (position: 19,025–19,624 bp and 106,895–107,700 bp in *C. muelleri* cpDNA) for *Chaetoceros* species.
