# Supplementary material for: Comparative Analysis of Chloroplast Genomes of Seven Chaetoceros Species Revealed Variation Hotspots and Speciation Time
Source: Front Microbiol. 2021 Nov 3;12:742554. doi: 10.3389/fmicb.2021.742554 (PMC8597025; doi:10.3389/fmicb.2021.742554)
Supplement: Supplementary file 1 [file Data_Sheet_1.docx]

**Table S1 Sampling station of *Chaetoceros* strains**

| **Species** | **Strains** | **Expedition dates** | **Location** | **Longitude** | **Latitude** |
| --- | --- | --- | --- | --- | --- |
| *C. muelleri* | CNS00047 | - | - | - | - |
| *C. costatus* | CNS00386 | July 2019 | The Changjiang Estuary, China | 122.865 | 30.363 |
| *C. socialis* | CNS00389 | August 2019 | The Jiaozhou Bay, China | 120.233 | 36.067 |
| *C. pseudo-curvisetus* | CNS00390 | August 2019 | The Jiaozhou Bay, China | 120.179 | 36.104 |
| *C. tenuissimus* | CNS00394 | May 2019 | The East China Sea, China | 121.342 | 21.38 |
| *C. laevisporus* | CNS00396 | July 2019 | The Changjiang Estuary, China | 122.865 | 30.363 |
| *C. curvisetus* | CNS00516 | July 2019 | The Jiaozhou Bay, China | 120.381 | 36.034 |

**Table S2. 95 shared protein-coding genes**

| **Category** | **Genes** |
| --- | --- |
| Photosystem Ⅰ | *psaA, psaB, psaD, psaF, psaJ, psaL* |
| Photosystem Ⅱ | *psbB*, *psbC*, *psbD*, *psbE*, *psbF*, *psbH*, *psbI*, *psbJ*, *psbK*, *psbL*, *psbN*, *psbT*, *psbV*, *psbX*, *psbY*, *psbZ* |
| Cytochrome b/f complex | *petA*, *petB*, *petD*, *petG*, *petL*, *petM*, *petN* |
| ATP synthase | *atpA*, *atpB*, *atpD*, *atpE*, *atpF*, *atpG*, *atpH*, *atpI* |
| RubisCO subunit | *rbcL*, *rbcS, rbcR* |
| RNA polymerase | *rpoA*, *rpoB*, *rpoC1*, *rpoC2* |
| Ribosomal proteins (SSU) | *rps2, rps3, rps4, rps5, rps7, rps9, rps10, rps11, rps12, rps13, rps14, rps16, rps17, rps18, rps20* |
| Ribosomal proteins (LSU) | *rpl1*, *rpl2*, *rpl3*, *rpl4*, *rpl5*, *rpl6*, *rpl11*, *rpl12*, *rpl13*, *rpl14*, *rpl16*, *rpl18*, *rpl19*, *rpl20*, *rpl23*, *rpl24*, *rpl29*, *rpl31*, *rpl32*, *rpl34*, *rpl35* |
| Other genes | *cbbX*, *ccs1*, *ccsA*, *chlI*, *clpC*, *dnaB*, *ftsH*, *groEL*, *secA*, *secG*, *secY*, *sufB*, *sufC*, *tatC*, *ycf3* |

**Table S3. The cpDNAs in the diatoms**

| Class | Species | Length | GC% | Access No. | Reference |
| --- | --- | --- | --- | --- | --- |
| Mediophyceae (22) | *Acanthoceras zachariasii* | 120,392 | 29.8 | MG755808 | (Yu et al., 2018) |
|  | *Attheya longicornis* | 129,565 | 31.1 | MG755798 | (Yu et al., 2018) |
|  | *Biddulphia biddulphiana* | 122,128 | 29.9 | MG755805 | (Yu et al., 2018) |
|  | *Biddulphia tridens* | 126,286 | 30.1 | MG755806 | (Yu et al., 2018) |
|  | *Cerataulina daemon* | 120,144 | 31.2 | KJ958484 | (Sabir et al., 2014) |
|  | *Chaetoceros muelleri* | 116,284 | 30.9 | NC_053621 | (Li and Deng 2021) |
|  | *Chaetoceros simplex* | 116,459 | 32.1 | NC_025310 | (Sabir et al., 2014) |
|  | *Cyclotella pseudostelligera* | 129,261 | 30.9 | MG755804 | (Yu et al., 2018) |
|  | *Cyclotella sp. L04_2* | 129,400 | 30 | KJ958480 | (Sabir et al., 2014) |
|  | *Cyclotella sp. WC03_2* | 129,498 | 30 | KJ958481 | (Sabir et al., 2014) |
|  | *Eunotogramma sp.* | 172,317 | 31.3 | MG755797 | (Yu et al., 2018) |
|  | *Leptocylindrus danicus* | 125,213 | 31.9 | KC509524 | (Ruck et al., 2014) |
|  | *Lithodesmium undulatum* | 122,660 | 31.1 | KC509525 | (Ruck et al., 2014) |
|  | *Odontella sinensis* | 119,704 | 31.8 | Z67753 | (Kowallik et al., 1995) |
|  | *Plagiogrammopsis vanheurckii* | 139,235 | 31.8 | MG755794 | (Yu et al., 2018) |
|  | *Roundia cardiophora* | 126,871 | 31 | KJ958483 | (Sabir et al., 2014) |
|  | *Skeletonema pseudocostatum* | 127,013 | 31.2 | MK372941 | (Hamedi et al., 2019) |
|  | *Thalassiosira oceanica* | 141,790 | 30.4 | GU323224 | (Markus et al., 2010) |
|  | *Thalassiosira pseudonana* | 128,814 | 30.7 | EF067921 | (Oudot-Le Secq et al., 2007) |
|  | *Thalassiosira weissflogii* | 127,601 | 30.8 | KJ958485 | (Sabir et al., 2014) |
|  | *Toxarium undulatum* | 141,681 | 29.8 | KX619437 | (Ruck et al., 2017) |
|  | *Pseudictyota dubia* | 120,381 | 32 | MG755801 | (Yu et al., 2018) |
| Bacillariophyceae (26) | *Fistulifera solaris* | 134,918 | 32.2 | AP011960 | (Tanaka et al., 2011) |
|  | *Gomphoneis minuta var. cassieae* | 119,630 | 32.7 | KY499654 | * NCBI |
|  | *Halamphora calidilacuna* | 150,738 | 32.3 | MK045451 | (Id et al., 2019) |
|  | *Plagiogramma staurophorum* | 201,816 | 29.6 | MG755792 | (Yu et al., 2018) |
|  | *Psammoneis obaidii* | 168,922 | 29.5 | MG755803 | (Yu et al., 2018) |
|  | *Seminavis robusta* | 150,905 | 30.9 | MH356727 | (Ruck et al., 2014) |
|  | *Synedra acus* | 116,251 | 30.6 | JQ088178 | (Galachyants et al., 2011) |
|  | *Asterionella formosa* | 121,238 | 30.6 | KC509519 | (Ruck et al., 2014) |
|  | *Asterionellopsis glacialis* | 146,024 | 31.2 | KC509520 | (Ruck et al., 2014) |
|  | *Astrosyne radiata* | 131,032 | 28.8 | MG755807 | (Yu et al., 2018) |
|  | *Cylindrotheca closterium* | 165,809 | 30.4 | KC509522 | (Ruck et al., 2014) |
|  | *Didymosphenia geminata* | 117,972 | 31.6 | KC509523 | (Ruck et al., 2014) |
|  | *Entomoneis sp.* | 122,056 | 32.4 | MG755800 | (Yu et al., 2018) |
|  | *Eunotia naegelii* | 152,906 | 28.7 | KF733443 | (Ruck et al., 2014) |
|  | *Fragilariopsis cylindrus* | 123,275 | 30.7 | NC_045244 | * NCBI |
|  | *Fragilariopsis kerguelensis* | 170,380 | 29.7 | LR812620 | * NCBI |
|  | *Halamphora americana* | 142,551 | 31.7 | MK045450 | (Id et al., 2019) |
|  | *Halamphora coffeaeformis* | 121,927 | 31.8 | MK045452 | (Id et al., 2019) |
|  | *Haslea nusantara* | 120,448 | 31.1 | MH681881 | (Prasetiya et al., 2019) |
|  | *Licmophora sp.* | 121,184 | 31.6 | MG755795 | (Yu et al., 2018) |
|  | *Nanofrustulum shiloi* | 160,994 | 31.6 | MN276191 | (Li et al., 2021) |
|  | *Nitzschia palea* | 119,116 | 32.8 | AP018511 | * NCBI |
|  | *Nitzschia palea* | 119,449 | 32.7 | MH113811 | (Crowell et al., 2019) |
|  | *Pseudo-nitzschia multiseries* | 111,539 | 31.4 | KR709240 | (Cao et al., 2016) |
|  | *Phaeodactylum tricornutum* | 117,369 | 32.5 | EF067920 | (Oudot-Le Secq et al., 2007) |
|  | *Phaeodactylum tricornutum* | 117,363 | 32.5 | MN937452 | * NCBI |
| Coscinodiscophyceae (7) | *Actinocyclus subtilis* | 119,120 | 29.4 | MG755799 | (Yu et al., 2018) |
|  | *Coscinodiscus radiatus* | 122,213 | 30.4 | NC_024081 | (Ruck et al., 2014) |
|  | *Guinardia striata* | 122,145 | 32.2 | MG755796 | (Yu et al., 2018) |
|  | *Proboscia sp.* | 138,249 | 32.6 | MG755791 | (Yu et al., 2018) |
|  | *Rhizosolenia fallax* | 125,283 | 30.2 | MG755802 | (Yu et al., 2018) |
|  | *Rhizosolenia imbricata* | 120,956 | 31.8 | KJ958482 | (Sabir et al., 2014) |
|  | *Rhizosolenia setigera* | 121,011 | 32.2 | MG755793 | (Yu et al., 2018) |

Note: *These chloroplast genomes are also publicly available in GenBank under mentioned accession numbers; NCBI, National Center for Biotechnology Information.

**Table S4. Calibration points used in the divergence time analysis by PAML**

| **Taxon1** | **Taxon2** | **Divergence time (Mya)** | **References** |
| --- | --- | --- | --- |
| *Ectocarpus siliculosus* | *Diatoms* | 176.0–202.0 | (Matari and Blair 2014) |
| *Rhizosolenia setigera* | *Skeletonema pseudocostatum* | 90.5-91.5 | (Damste et al., 2004) |
| *Pseudo-nitzschia multiseries* | *Fragilariopsis cylindrus* | 10.0-35.3 | (Matari and Blair 2014) |

Cao, M., Yuan, X.L., Bi, G. (2016). Complete sequence and analysis of plastid genomes of *Pseudo-nitzschia multiseries* (Bacillariophyta). *Mitochondrial DNA A DNA Mapp Seq Anal*. 27 (4):2897-2898.

Crowell, R.M., Nienow, J.A., Cahoon, A.B. (2019). The complete chloroplast and mitochondrial genomes of the diatom Nitzschia palea (Bacillariophyceae) demonstrate high sequence similarity to the endosymbiont organelles of the dinotom Durinskia baltica. *J Phycol*. 55 (2):352-364.

Damste, J.S., Muyzer, G., Abbas, B., Rampen, S.W., Masse, G., Allard, W.G. et al. (2004). The Rise of the Rhizosolenid Diatoms. *SCIENCE*. 304.

Galachyants, Y.P., Morozov, A.A., Mardanov, A.V., Beletsky, A.V., Ravin, N.V., Petrova, D.P. et al. (2011). Complete Chloroplast Genome Sequence of Freshwater Araphid Pennate Diatom Alga *Synedra acus* from Lake Baikal. *International Journal of Biology*. 4 (1).

Hamedi, C., Gastineau, R., Lemieux, C., Turmel, M., Witkowski, A., Hamed, M.B. (2019). Complete chloroplast genome of the diatom <i>Skeletonema pseudocostatum</i> from the Western Mediterranean coast of Algeria. *Mitochondrial Dna Part B*. 4 (1):1091-1092.

Id, S., Id, K., Pogoda, C.S., Stepanek, J.G., Kociolek, J.P. (2019). Extensive chloroplast genome rearrangement amongst three closely related Halamphora spp. (Bacillariophyceae), and evidence for rapid evolution as compared to land plants. *PLoS One*. 14 (7):e0217824.

Kowallik, K.V., Stoebe, B., Schaffran, I., Kroth-Pancic, P., Freier, U. (1995). The chloroplast genome of a chlorophyll a+c -containing alga, Odontella sinensis. *Plant Molecular Biology Reporter*. 13 (4):336-342.

Li, C., Gastineau, R., Turmel, M., Witkowski, A., Lemieux, C. (2021). Mitochondrial DNA Part B Complete chloroplast genome of the tiny marine diatom Nanofrustulum shiloi (Bacillariophyta) from the Adriatic Sea.

Li, Y., Deng, X. (2021). The complete chloroplast genome of the marine microalgae *Chaetoceros muellerii* (Chaetoceroceae). *Mitochondrial DNA B Resour*. 6 (2):373-375.

Markus, Lommer, Alexandra-Sophie, Roy, Markus, Schilhabel et al. (2010). Recent transfer of an iron-regulated gene from the plastid to the nuclear genome in an oceanic diatom adapted to chronic iron limitation. *BMC Genomics*. 11 (1):718-718.

Matari, N.H., Blair, J.E. (2014). A multilocus timescale for oomycete evolution estimated under three distinct molecular clock models. *Bmc Evolutionary Biology*. 14 (1):101-101.

Oudot-Le Secq, M.P., Grimwood, J., Shapiro, H., Armbrust, E.V., Bowler, C., Green, B.R. (2007). Chloroplast genomes of the diatoms Phaeodactylum tricornutum and Thalassiosira pseudonana: comparison with other plastid genomes of the red lineage. *Mol Genet Genomics*. 277 (4):427-439.

Prasetiya, F.S., Gastineau, R., Poulin, M., Lemieux, C., Leignel, V. (2019). Haslea nusantara (Bacillariophyceae), a new blue diatom from the Java Sea, Indonesia: morphology, biometry and molecular characterization. *Plant Ecology and Evolution*. 151 (2):188-202.

Ruck, E.C., Linard, S.R., Nakov, T., Theriot, E.C., Alverson, A.J. (2017). Hoarding and horizontal transfer led to an expanded gene and intron repertoire in the plastid genome of the diatom, Toxarium undulatum (Bacillariophyta). *Curr Genet*. 63 (3):499-507.

Ruck, E.C., Nakov, T., Jansen, R.K., Theriot, E.C., Alverson, A.J. (2014). Serial gene losses and foreign DNA underlie size and sequence variation in the plastid genomes of diatoms. *Genome Biol Evol*. 6 (3):644-654.

Sabir, J.S., Yu, M., Ashworth, M.P., Baeshen, N.A., Baeshen, M.N., Bahieldin, A. et al. (2014). Conserved gene order and expanded inverted repeats characterize plastid genomes of Thalassiosirales. *PLoS One*. 9 (9):e107854.

Tanaka, T., Fukuda, Y., Yoshino, T., Maeda, Y., Muto, M., Matsumoto, M. et al. (2011). High-throughput pyrosequencing of the chloroplast genome of a highly neutral-lipid-producing marine pennate diatom, Fistulifera sp. strain JPCC DA0580. *Photosynth Res*. 109 (1-3):223-229.

Yu, M., Ashworth, M.P., Hajrah, N.H., Khiyami, M.A., Sabir, M.J., Alhebshi, A.M. et al. (2018) Evolution of the Plastid Genomes in Diatoms. In. *Advances in Botanical Research*. pp 129-155.
