# Supplementary figures and images for: Comparative Analysis of Chloroplast Genomes of Seven Chaetoceros Species Revealed Variation Hotspots and Speciation Time
Source: Front Microbiol. 2021 Nov 3;12:742554. doi: 10.3389/fmicb.2021.742554 (PMC8597025; doi:10.3389/fmicb.2021.742554)

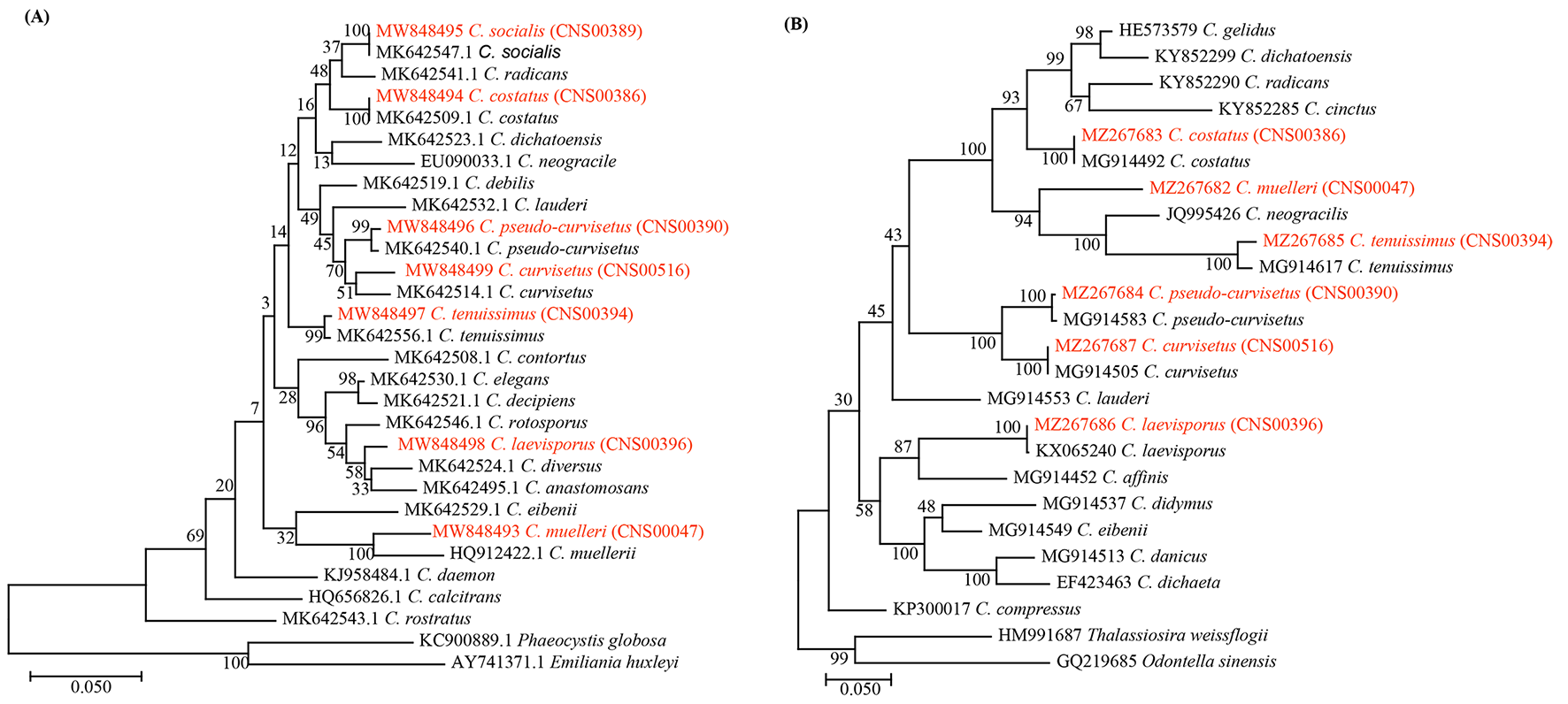

Supplement: Supplementary Figure 1 — The phylogenetic analysis of Chaetoceros species using full length of (A) rbcL gene and (B) partial 28S rDNA. [file Image_1.TIF]

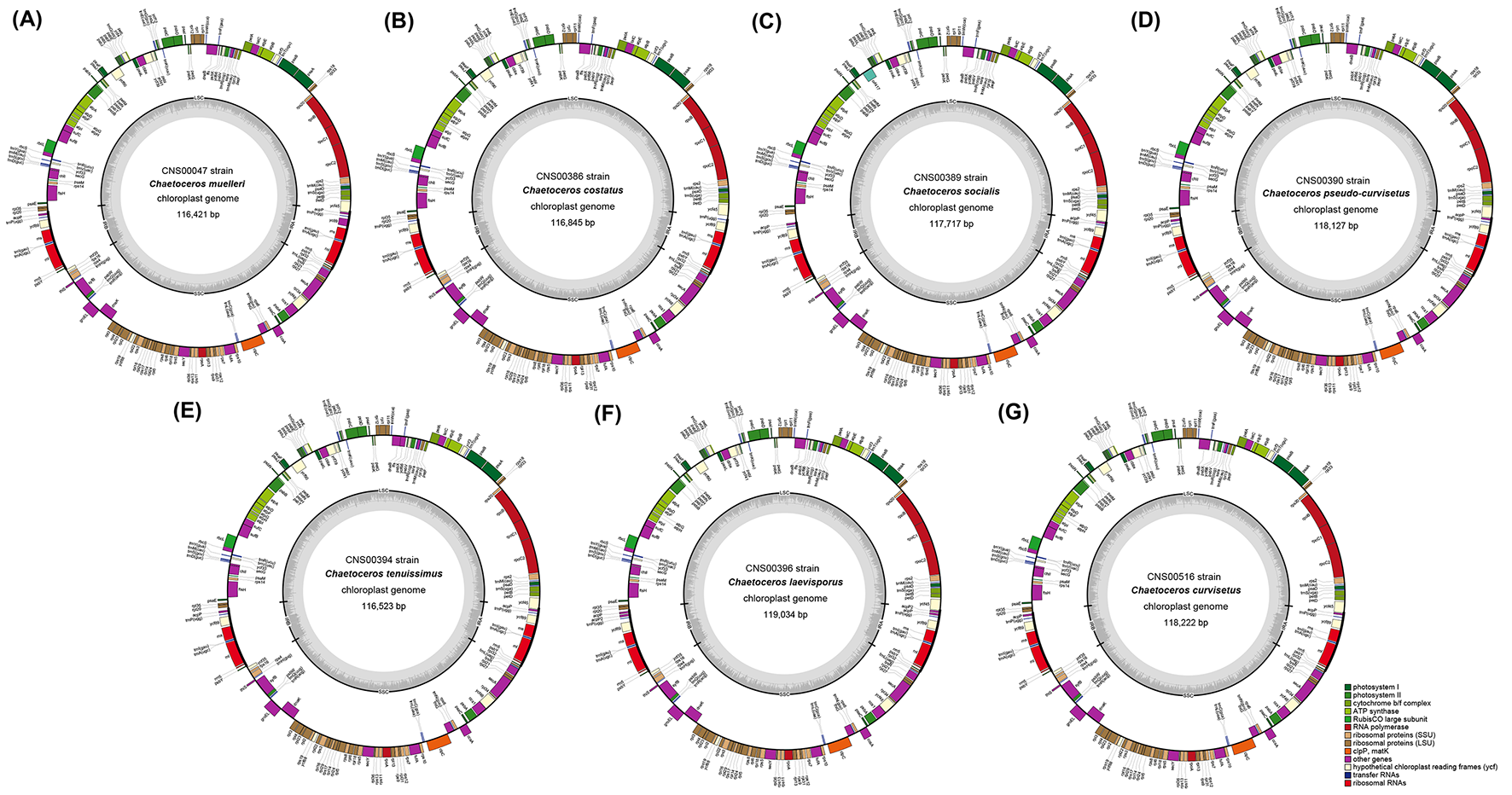

Supplement: Supplementary Figure 2 — Gene structural map of the seven Chaetoceros cpDNAs. (A) C. muelleri (strain CNS00047), (B) C. costatus (strain CNS00386), (C) C. socialis (strain CNS00389), (D) C. pseudo-curvisetus (strain CNS00390), (E) C. tenuissimus (strain CNS00394), (F) C. laevisporus (strain CNS00396), and (G) C. curvisetus (strain CNS00516). [file Image_2.TIF]

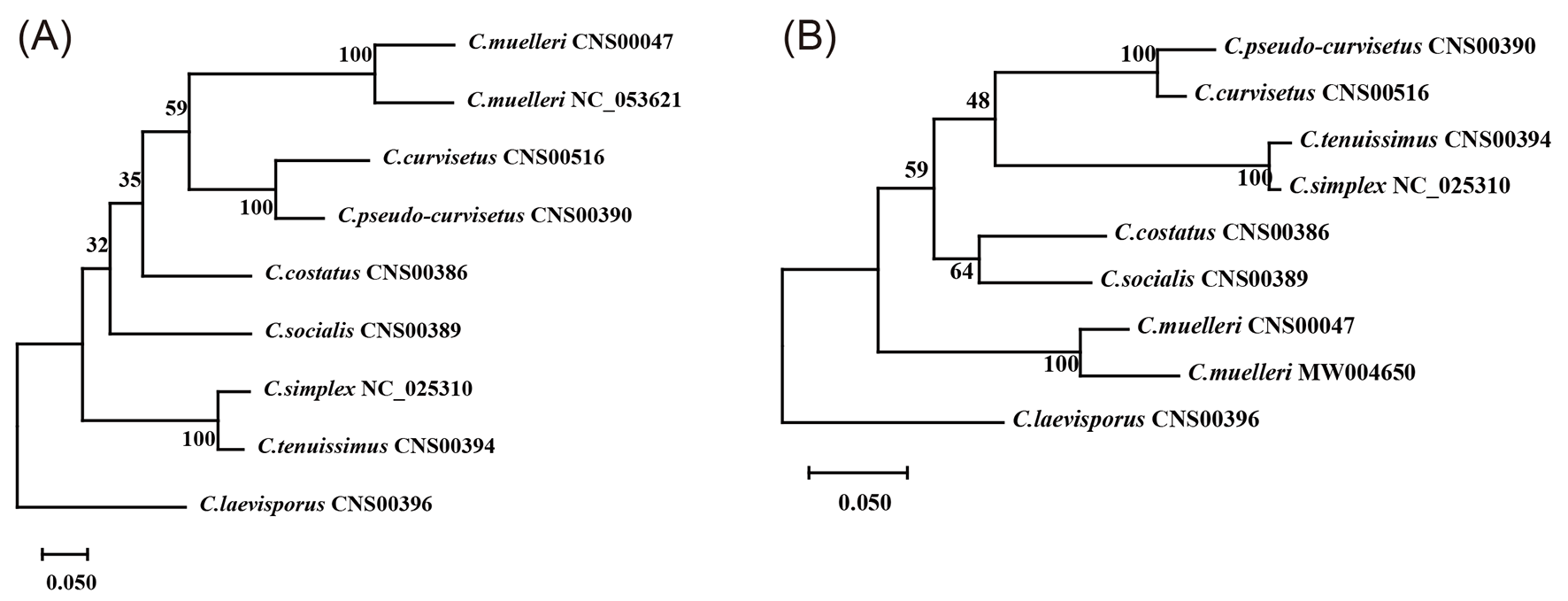

Supplement: Supplementary Figure 3 — Phylogenetic trees constructed using different hotspots regions. (A) Position: 19,025–19,624 bp in C. muelleri cpDNA. (B) 106,895–107,700 bp in C. muelleri cpDNA. [file Image_3.TIF]

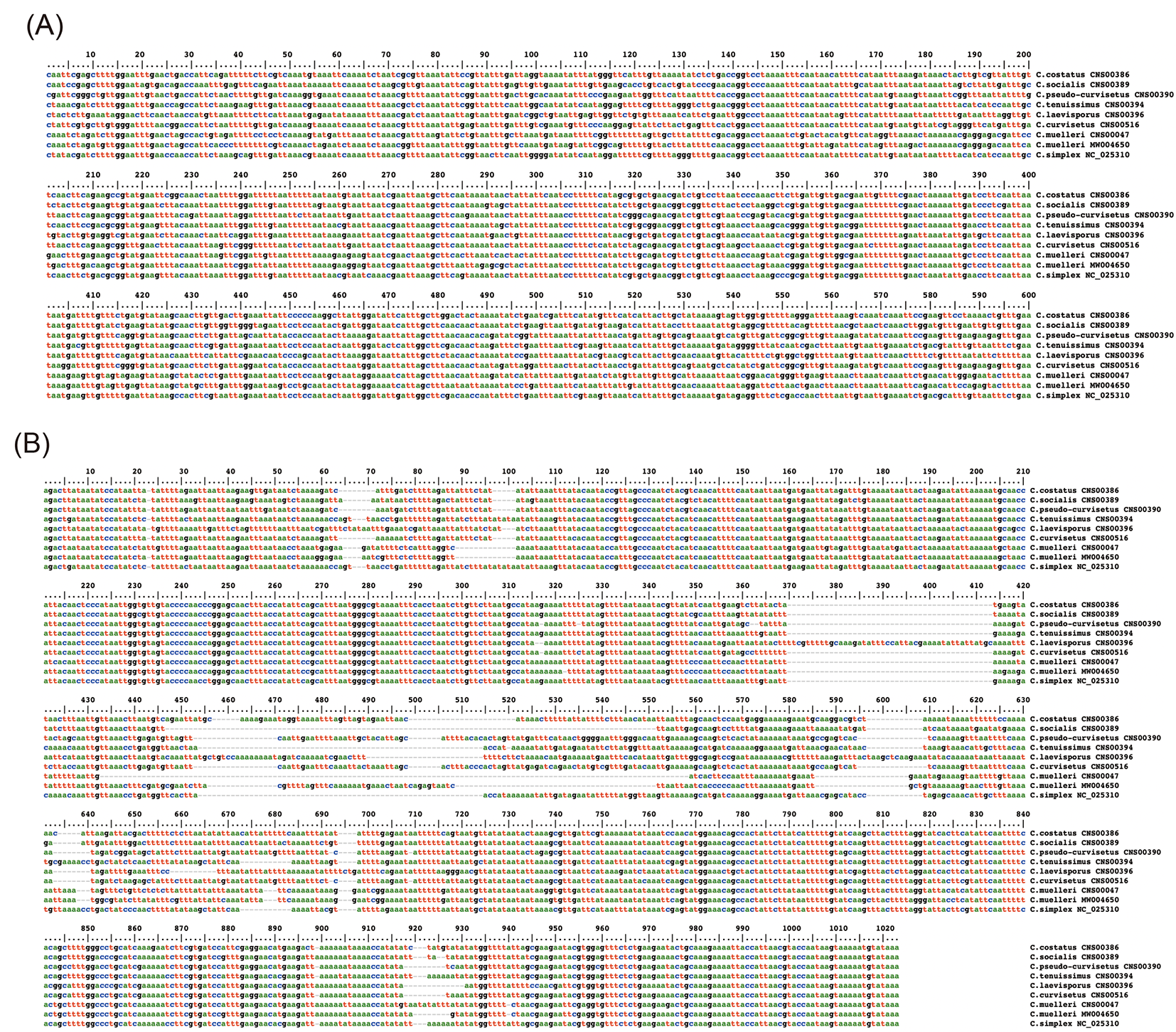

Supplement: Supplementary Figure 4 — (A,B) The DNA alignment information of hotspots region (position: 19,025–19,624 bp and 106,895–107,700 bp in C. muelleri cpDNA) for Chaetoceros species. [file Image_4.TIF]
